# Gender Differences in the COVID-19 Pandemic Risk Perception, Psychology, and Behaviors of Spanish University Students

**DOI:** 10.3390/ijerph18083908

**Published:** 2021-04-08

**Authors:** Stephanie Rodriguez-Besteiro, José Francisco Tornero-Aguilera, Jesús Fernández-Lucas, Vicente Javier Clemente-Suárez

**Affiliations:** 1 Faculty of Sports Sciences, Universidad Europea de Madrid, Tajo Street, s/n, 28670 Madrid, Spain; stephanie.rodriguez@universidadeuropea.es (S.R.-B.); josefrancisco.tornero@universidadeuropea.es (J.F.T.-A.); 2 Studies Centre in Applied Combat (CESCA), 45007 Toledo, Spain; 3 Applied Biotechnology Group, European University of Madrid, c/ Tajo s/n, Villaviciosa de Odón, 28670 Madrid, Spain; jesus.fernandez2@universidadeuropea.es; 4 Grupo de Investigación en Ciencias Naturales y Exactas, GICNEX, Universidad de la Costa, CUC, 080002 Barranquilla, Colombia; 5 Grupo de Investigación en Cultura, Educación y Sociedad, Universidad de la Costa, 080002 Barranquilla, Colombia

**Keywords:** gender differences, COVID-19, students, risk perception, anxiety, personality

## Abstract

The actual COVID-19 pandemic scenario has generated a context of uncertainty, helplessness, and inequality. Yet, the perception of COVID-19 risk has influenced nutritional, psychological, and physical activity patterns depending on gender. We conducted the present research with the aim of studying gender differences of university students in the perceived risk of the COVID-19 pandemic, and in psychological, nutritional, oral health, and physical activity habits. To reach the study’s aim, 300 volunteer university students completed an online questionnaire which analyzed variables of perceived risk of the COVID-19 pandemic, psychological profiles, and nutritional, oral health, and physical activity habits. Results showed that females presented a higher perception of danger to the COVID-19 virus than males but showed no differences in how the pandemic has affected personal lives. Females showed higher values of anxiety, conscientiousness, neuroticism, and openness to experience, while males presented higher values of extraversion. Nutritionally, males presented greater consumption of soft drinks, meat, and pasta or rice, and lower buccal hygiene. Yet, no differences were found regarding physical activity patterns. Results from the present study could be used by various educational institutions to implement multidisciplinary interventions to reduce the stress and risk perception.

## 1. Introduction

Originating in Wuhan (Hubei, China) in December 2019 as a cluster of unexplained cases of pneumonia, the World Health Organization classified the SARS-Cov-2 outbreak as a pandemic in March 2020, affecting multiple countries, with more than 110 million confirmed cases and more than 2.5 million deaths [1]. On 26 February 2020, the first case of COVID-19 was detected in Spain. Due to the large increase in the number of cases, on 14 March, the Spanish government declared a state of alarm throughout the country. Beyond impacting millions of lives around the world, the pandemic has dealt a blow to the economy on a global level. The COVID-19 health crisis has posed a complex scenario for economy not only because of the shock it has produced, but also because its repercussions will be significant [2]. The world economy is facing its greatest challenge since the Great Recession. The state of alarm in Spain has resulted in the confinement of millions of people and, for this reason, the Spanish economy was forced to establish urgent measures to avoid the paralysis of both public and private administrative activity. In this way, many companies were forced to implement teleworking quickly so that their employees could continue to carry out their duties from home. Similarly, universities also moved 75% of their students [3] to online learning so that they could continue their studies [4]. However, not all companies have been able to adapt to this new modality, so they have been forced to permanently or temporarily suspend all or part of their activity, exercising Temporary Employment Regulation Files or on many occasions to dismiss their employees.

Because of the interactions between biological factors and social determinants, including gender stereotypes, differences and roles, social stigma, and social autonomy [5], inequities are expected to appear in the context of COVID-19. Indeed, COVID-19 has affected males and females differently, presenting higher fatality rates, a worse prognosis, and a higher risk of death in males [6]. Yet, despite fatality rates, females have a higher prevalence and severity of anxiety, depression, and acute stress symptoms [7]. However, females have experienced a greater number of psychological alterations that can be associated with isolated symptoms and complex disorders, which are related to a deterioration in functionality and the development of anxiety, insomnia, depression, or post-traumatic stress disorder (PTSD). In addition, gender moderates the relationship between emotional disturbances (e.g., psychological distress) and personal strengths such as resilience and social support in students. Thus, differences in psychometric and emotional profiles are key elements to understand the striking differences between males and females regarding COVID-19 beliefs and behaviors.

In this line, the authors hypothesize that females are more likely to take the pandemic seriously. In March, 59% of female respondents considered COVID-19 to be a very serious health problem compared to 49% of males. In mid-April, both numbers decreased, but the gender difference remained: 40% of females still saw the virus as a very serious risk compared to 33% of males. This difference is present among studied countries [8]. Indeed, the authors postulate that gender differences regarding perception risk are echoed in behavioral differences between male and female leaders. Countries which are led by females have responded with greater effectiveness to the pandemic than countries led by males [9]. For example, Germany, Iceland, New Zealand, and Denmark, which have female leaders, have used a more democratic and inclusive style of leadership, with decisive and clear communication strategies. Meanwhile, countries with male leaders such as the US, Brazil, and the UK have experienced the worst COVID-19 outcomes [9].

Yet, one of the most affected by the COVID-19 pandemic groups are students, since their welfare and mental health is threatened. Previous research on COVID-19’s psychological effect on university students indicates that the economic situation, as well as delays in academic activities, are risk factors for developing anxiety, with depressive symptoms, stress, and anxiety being the most commonly identified psychological effects [10]. When compared to other collectives, such as professors, students seem to present higher scores of stress and anxiety [11], with females presenting higher ratios and a growing and greater prevalence of depression among male students [12]. However, the psychological and emotional profiles and the behavioral responses depend greatly on both contextual and multifactorial factors such as nutritional status, oral health, and the amount of physical exercise [13]. All of these factors are influenced by gender [14], and previous authors have remarked that these factors may be influenced by the pandemic situation (-) and lockdown (-).

In this line, researchers have established an association between the way people eat and their mood. Thus, eating patterns can affect the way people feel [15]. During the period of confinement, nutritional habits changed dramatically in parallel with the increase in anxiety and stress values among the population [16]. Previous authors have found that the most frequent changes related to an increased consumption of fruit (27%), eggs (25.4%), legumes (22.5%), vegetables (21%), and fish (20%), and a reduced consumption of processed meats (35.5%) and sugary drinks (32.8%), with clear differences according to age and gender. Physical activity can be a contextual factor for the psychological profile. Students who are physically active tend to have a healthier and more balanced diet than those who are not physically active. University students practice an average 40 min of physical activity per day, being significantly higher in males than in females [17]. Along these lines, male university students tend to opt for sporting activities in their leisure time, while females give greater importance to other social activities and personal hobbies in detriment of physical activity [18]. Few studies have focused on gender differences regarding the impact of the COVID-19 outbreak countries like Spain, where mortality remains one of the highest worldwide, especially when considering a wide range of multifactorial variables. Thus, we conducted the present research with the aim of studying gender differences in university student regarding the perceived risk of the COVID-19 pandemic and in psychological, nutritional, oral health, and physical activity habits. The initial hypotheses were: (i) There are gender differences in the perceived risk of the COVID-19 pandemic, and (ii) there are gender differences in the psychological, nutritional, oral health, and physical activity habits of students.

## 2. Materials and Methods

In the current study, 300 university students residing in Spain, aged between 17 and 51 years (according to the sample obtained), were interviewed via online questionnaire in a period of 3 months, from October 2020 to December 2020. Our inclusion criteria were: Enrollment in the current academic year, currently living in Spain, and either graduate or undergraduate students from any field/area of expertise. In order to prevent double responses from the same person, students had to include their Student ID, which was required to match with the university database. Furthermore, data were considered strictly confidential. This research complied with the Helsinki declarations (revised in Brazil, 2013), on human research and was approved by the University Ethics Committee (CIPI/18/074).

All of the participants digitally signed a consented participation where the aims and procedure of the study was explained. To reach the aim of the present research, a cross-sectional study was developed. The following parameters were analyzed.

### 2.1. Sociodemographic Factors

Age (years), height (cm), weight (kg), and Body Mass Index (BMI, Kg/m^2^) were analyzed, along with the degree of compliance with the confinement due to the COVID-19 crisis using a Likert scale, where 0 means the least and 10 the most. The question, “How many people you have lived with during the confinement?” was measured on a self-perception scale, indicating the number of people with which the student lived.

### 2.2. Economic Variables

We analyzed whether the university students performed any type of paid work. If so, we then asked whether this had been affected by the COVID-19 crisis. The options were: Not affected, reduced working hours and income reduced, and job loss.

### 2.3. Psychological Profile

We analyzed the students’ perceived danger of the COVID-19 virus using a Likert scale from 0 to 10, where 0 means the least and 10 is the most. A Likert scale was also used to measure how the COVID-19 crisis has affected the participant personally, where 0 means the least and 10 the most. A reduced version of the Spanish version of the Big Five Inventory [19] was used to measure personality traits, including openness to experience, conscientiousness, extraversion, agreeableness, and neuroticism. The reduced version is composed of 10 items that are answered on a 5-point Likert scale, where 1 means completely disagree and 5 means completely agree. A reduced version of the Spanish version of Spielberger State-Trait Anxiety Inventory [20], composed of 6 items assessing anxiety that are answered on a 4-point Likert scale where 1 means not at all and 4 means very much, was used to measure anxiety. The Spanish version of the Acceptance and Action Questionnaire II [21] was used to analyze the experiential avoidance or psychological inflexibility through 7 items answered by a 7-point Likert scale, where 0 means never true and 7 means always true. The Spanish version of the UCLA Loneliness Scale [22] was used to scale measures loneliness. In the present study, we used a condensed version which consists of 3 items answered by a 3-point Likert scale, where 1 means never and 3 means frequently. The Spanish version of Zung Depression Scale [23] was used to measure depression in relation to the COVID-19 crisis. The Zung Depression Scale uses a self-applied scale for depression, which has a sensitivity and specificity greater than 80% and consists of 20 items formulated in positive and negative terms. Somatic and cognitive symptoms are highly relevant, with 8 items for each group. The scale also includes 2 items referring to mood and 2 to psychomotor symptoms.

### 2.4. Health-Related Factors

Hours of sleep per day were measured on a self-perception scale, indicating the number of hours the student sleep per day. The quality of the parcipants’ last sleep was measured using a Likert scale, where 1 means very poor sleep quality and 10 means very good sleep quality. Average number of steps per day in the last week was measured on a self-perception scale, indicating the number of steps the student had taken in the last week. Nutritional habits were analyzed using an adapted previously used questionnaire. The first 2 questions were related to eating habits. The rest of questions to the consumption frequency of different food groups, including fish, vegetables, legumes, meat, fast food, soft drinks, in which answers ranged from “less than two per week” to “seven or more per week.” For oral health, a previously used questionnaire consisting of 4 items related to oral health was used. For the first question (“How many times a day do you brush your teeth?”), the answers ranged from “none” to “more than four per day.” For the question “Do you smoke?”, answers ranged from “no” to “more than five cigarettes per day.” The rest of questions were answered by “yes,” “sometimes,” or “no.” Physical activity habits were measured with a questionnaire used in line with previous research. We evaluated the psychophysiological stress response in high psychologically demanding contexts using a questionnaire which included the items: “Did you do any physical activity in the last 7 days?”, “If so, time in minutes of cyclic and/or aerobic activity (cycling, treadmill, Zumba) adding up all the sessions of the 7 days”, “If so, time in minutes of activity with self-loads (sit-ups, push-ups, squats...) or weights (gym machines, weights...) adding up all the sessions of the 7 days.”

### 2.5. Statistical Analysis

Statistical analyses were analyzed using the Statistical Package for the Social Sciences (SPSS) version 24.0 (SPSS Inc., Chicago, IL, USA). Descriptive statistics (mean and standard deviation) were calculated for each variable. Kolmogorov–Smirnov tests were performed to analyze normality and homogeneity of each variable. To analyze gender differences in sociodemographic, academic, and psychological variables, an independent T test was conducted. To analyze gender differences in economic, health-related, and oral health variables, the Chi-square test was used. The level of significance was set at *p* ≤ 0.05.

## 3. Results

Data are presented as mean ± standard deviation. Anthropometrical differences were found regarding height, weight, and BMI (Table 1).

Regarding economic variables, no gender differences were found in how the COVID-19 pandemic has affected employment (Table 2).

According to the academic variables, no gender differences were found in how the COVID-19 pandemic has affected studies.

According to the psychological profile, females showed a higher perception of danger to the COVID-19 virus than males. Females presented higher values in conscientiousness, neuroticism, openness to experience, and stress than males. However, males presented higher values of extraversion than females. Yet, no gender differences were seen for psychological traits such as depression, loneliness, and experiential avoidance (Table 3). Reliability was estimated through Cronbach’s alpha, obtaining 0.729 for Big Five factors, 0.810 for the Acceptance and Action Questionnaire II (AAQII), 0.870 for the UCLA Loneliness Scale (UCLA), 0.854 for the Spielberger State-Trait Anxiety Inventory (STAI), and 0.793 for the Zung Depression Scale (ZUNG).

Regarding the health-related factors, males presented a higher weekly consumption of soft drinks, meat, and pasta or rice than females. Females showed higher values in daily tooth brushing and dry mouth than males. No gender differences were found in the physical activity habits analyzed (Table 4).

## 4. Discussion

The aim of the present research was to study gender differences among university students regarding the perceived risk of the COVID-19 pandemic and in psychological, nutritional, oral health, and physical activity habits. The initial hypothesis was partially confirmed, since female students showed higher scores on the level of perceived risk of the COVID-19 pandemic than male students. However, significant differences between genders were found in some psychological and nutritional variables but not in oral health and physical activity variables.

In the present study, females presented higher perceived risks level of the COVID-19 pandemic than males. Authors have suggested that there a is a gender difference in the psychological experience, somatization, and impact of the COVID-19 pandemic and the emotions it provokes, suggesting that women are more emotionally vulnerable to the effects of COVID-19 context than men [24]. This may be related to the greater levels of state-trait anxiety reached in this study, where females presented higher levels than males in lin with previous literature [25]. This may also explain the greater emotional vulnerability of females [26]. Indeed, there are also gender differences in stress coping among university students [27], where females have shown greater stress and lower stress coping abilities than male [28], thus supporting our results.

The psychometric profile and personality trait differences between genders may explain the stronger influence of perceived risk and anxiety in females. Within these personality traits, our data suggest that male students have higher levels of extraversion than females, while females present higher values in conscientiousness and neuroticism, which is in lineprevious research conducted in female professors [29]. The present data suggest that females have greater openness to experience, contrary to the results of Castañeiras et al. (2006), where males showed higher levels of openness to experience than females [30]. However, these differences could be attributed to the difference in the sociocultural context (Latin America-Europe), as well as the context of the sample, since our sample was students.

Regarding the nutritional profile, no gender differences were found among the consumption of fruit, legume, or vegetables, which is contrary to previous studies. Authors have suggested that male’s poorer nutrition knowledge explains a significant part of their lower intake of fruit and vegetables [31], with a tendency for fat and protein rich foods breweries as beer, spirits, and sweet carbonated drinks [32], in line with our data. Yet, it has been reported that students have poor nutrition habits [33], reflecting a significant gender difference in weight status with the percentage of overweight/obese males being more than double that of females [34]. However, no gender differences were seen in the present study as in previous research in the COVID-19 pandemic [35].

According to oral health profile, females showed significantly higher values for daily tooth brushing, dry mouth, and gastritis than males. This high frequency in daily tooth brushing is consequent with previous research and may be related to the higher values of neuroticism and conscientiousness shown by females [36]. However, no significant relationships have been found between toothbrushing and psychological factors [37]. In the same way, dry mouth or lack of saliva has also been related to increased stress perception and the somatization of anxiety and depression, conforming to a psych emotional profile and stress perception of the analyzed female sample. Thus, a relationship was found between stress and oral health, where females tended to suffer more than males despite the high frequency of brushing, which coincides with the literature found in other groups such as teachers [38].

Regarding the physical activity profile, no gender differences were found, which is in line with previous literature among university students [39]. Yet, values or physical exercise were still down considering the minimum requirements of daily/weekly physical exercise, which is in line with data found in gender and university students in previous research [40,41]. Indeed, authors have suggested that students who do not engage in physical exercise or sport present greater stress reactions [42]. Yet, authors have suggested that younger students present better performance in physical exercise, academics, and work, demonstrating a good lifestyle compared to older students [43].

The multifactorial analysis of factors related to the perception of risk level of COVID-19 may be a useful tool to measure the associated stress in university students to explain and prevent the psychological consequences of the COVID-19 pandemic. In addition, the use of questionnaires allows significant information to be collected in a short period of time. Knowledge of these related factors could be used by various educational institutions to implement multidisciplinary interventions to reduce this perception and, thus, students’ stress in the face of the virus. The present research also presents some limitations, with the main limitation being the lack of biological measurement due to COVID-19 and the impossibility of measuring stress hormones (cortisol, adrenaline, alpha amylase…). Other limitations were that anthropometrical data were self-declared, which may lead to a serious risk of bias. However, since this was an online questionnaire, no other further methods of evaluations were possible. Future studies may address this issue. As a future research line, we propose analyzing the influence of cultural differences in the levels of perceived danger from the COVID-19 virus. In addition, this study could be extended to other degrees, as well as to other educational levels such as primary and secondary school.

## 5. Conclusions

We can conclude that female university students presented higher levels of perceived danger from the COVID-19 virus than male university students. Males showed higher levels of extraversion than females, but females showed higher levels of conscientiousness, neuroticism, and openness to experience. Females showed higher levels of perceived anxiety than males. Regarding the nutritional profile, males showed a higher frequency of consumption of soft drinks, meat, pasta, or rice. Regarding oral health, females showed a higher number of times they brushed their teeth, as well as a higher frequency of dry mouth or lack of saliva. In the physical activity profile, no significant results were found in either gender.

The multifactorial analysis of factors related to the perception of the level of danger to COVID-19 may be a useful tool to measure the associated stress in university students to explain and prevent the psychological consequences of the COVID-19 pandemic. In addition, the use of questionnaires allows significant information to be collected in a short period of time. Awareness of these related factors could be used by various educational institutions to implement multidisciplinary interventions to reduce this perception and, thus, students’ stress in response to the virus.

## Figures and Tables

**Table 1:** Gender differences in sociodemographic factors.

Variable	Male	Female	*t*	*p*
Age (yrs)	23.86 ± 5.45	24.40 ± 6.95	0.711	0.477
Height (cm)	178.17 ± 6.46	162.45 ± 17.10	−10.158	0.000
Weight (Kg)	79.98 ± 55.29	59.33 ± 8.44	−4.082	0.000
Body Mass Index (BMI)	23.65 ± 2.93	21.92 ± 2.87	−4.787	0.000
Degree of confinement compliance due to the COVID-19 crisis	8.65 ± 1.69	8.91 ± 1.68	1.218	0.224
How many people have you lived with in confinement?	2.74 ± 1.24	2.57 ± 1.19	−1.141	0.255

**Table 2:** Gender differences in economic variables.

Variable	Male	Female	Chi-Squared	*p*
Do you perform any paid work?	1.46 ± 0.65	1.54 ± 0.71	0.469	0.333
Regarding your work. have you been affected by the COVID-19 crisis?	1.46 ± 0.65	1.59 ± 0.67	0.272	0.177

**Table 3:** Gender differences in psychological profiles.

Variable	Male	Female	*t*	*p*
Level of perceived danger in the COVID-19 Pandemic	6.49 ± 2.03	7.20 ± 1.65	3.089	0.002
Extraversion	5.88 ± 1.71	5.27 ± 1.69	−2.906	0.004
Agreeableness	6.24 ± 1.55	6.56 ± 1.577	1.673	0.096
Conscientiousness	6.39 ± 1.89	7.08 ± 1.69	3.132	0.002
Neuroticism	5.74 ± 2.12	6.72 ± 2.27	3.609	0.000
Openness to experience	6.96 ± 1.69	7.48 ± 1.76	2.471	0.014
AAQII	23.36 ± 8.90	24.22 ± 11.04	0.702	0.483
UCLA	4.47 ± 1.76	4.47 ± 1.61	−0.033	0.974
ZUNG	41.73 ± 4.47	42.70 ± 5.23	31.945	0.234

AAQII (Acceptance and Action Questionnaire II); UCLA (UCLA Loneliness Scale); STAI (Spielberger State-Trait Anxiety Inventory); ZUNG (Zung Depression Scale).

**Table 4:** Gender differences in the health-related factors.

Variable	Male	Female	Chi-Squared	*p*
How many meals did you take on average during your confinement?	4.28 ± 1.25	4.50 ± 1.39	13.168	0.155
How many glasses of water do you drink per day?	4.89 ± 1.34	4.80 ± 1.43	5.262	0.385
Juices	1.63 ± 0.97	1.52 ± 0.90	2.458	0.483
Alcoholic Beverage	1.06 ± 0.27	1.04 ± 0.23	1.153	0.562
Fermented beverage	1.37 ± 0.70	1.30 ± 0.60	1.707	0.635
Soft drinks	1.58 ± 0.87	1.38 ± 0.69	4.118	0.042
Energy Drink	1.16 ± 0.44	1.12 ± 0.43	3.730	0.155
Fruit	2.68 ± 1.06	2.83 ± 1.01	3.230	0.357
Bakery/Sweets	1.72 ± 0.83	1.76 ± 0.89	2.931	0.402
Meat	2.87 ± 0.75	2.28 ± 0.95	34.075	0.000
Fish	2.00 ± 0.79	1.87 ± 0.81	6.846	0.077
Legume	2.21 ± 0.82	2.00 ± 0.84	11.721	0.008
Pasta or rice	2.69 ± 0.80	2.16 ± 0.89	26.040	0.000
Vegetables	2.55 ± 0.95	2.75 ± 0.98	3.826	0.281
Bread	2.70 ± 1.14	2.61 ± 1.18	1.669	0.644
Fast food	1.37 ± 0.63	1.28 ± 0.53	2.596	0.458
Do you smoke?	1.18 ± 0.60	1.29 ± 0.79	6.369	0.095
Do you suffer from gastritis or heartburn?	2.17 ± 0.49	2.20 ± 0.48	0.617	0.735
How many times do you brush your teeth per day?	2.39 ± 0.87	2.71 ± 0.80	3.078	0.002
Does your mouth often feel dry as if it lacks saliva?	2.11 ± 0.53	2.25 ± 0.59	2.057	0.041
Minutes of cyclic and/or aerobic activity	276.97 ± 243.24	227.77 ± 239.74	45.267	0.227
Minutes of activity with self-loading or weights	217.68 ± 209.87	236.36 ± 254.01	35.254	0.760

## Data Availability

All data are presented in the manuscript.

## References

[1] WHO—World Health Organization (2020). Coronavirus Disease (COVID-19) Dashboard.

[2] Torres R., Fernández M.J. (2020). La política económica española y el COVID-19. Funcas Cuad. Inf. Económica.

[3] Secretaría General de Universidades (2019). Datos y Cifras del Sistema Universitario Español.

[4] Díaz A.L., Prados J.S.F., Canos V.F., Martínez A.M.M. (2020). Impactos del confinamiento por el COVID-19 entre universitarios: Satisfacción Vital, Resiliencia y Capital Social Online. RISE.

[5] Hou F., Bi F., Jiao R., Luo D., Song K. (2020). Gender differences of depression and anxiety among social media users during the COVID-19 outbreak in China: A cross-sectional study. BMC Public Health.

[6] Spagnolo P.A., Manson J.E., Joffe H. (2020). Sex and Gender Differences in Health: What the COVID-19 Pandemic Can Teach Us. Ann. Intern. Med..

[7] Liu N., Zhang F., Wei C., Jia Y., Shang Z., Sun L., Liu W., Wu L., Sun Z., Zhou Y. (2020). Prevalence and predictors of PTSS during COVID-19 outbreak in China hardest-hit areas: Gender differences matter. Psychiatry Res..

[8] Galasso V., Pons V., Profeta P., Becher M., Brouard S., Foucault M. (2020). Gender differences in COVID-19 related attitudes and behavior: Evidence from a panel survey in eight OECD countries. Proc. Natl. Acad. Sci. USA.

[9] Garikipati S., Kambhampati U. (2020). Women Leaders are Better Fighting at the Pandemic.

[10] Cobo-Rendón R., Vega-Valenzuela A., García-Álvarez D. (2020). Consideraciones institucionales sobre la Salud Mental en estudiantes universitarios durante la pandemia de COVID-19. CienciAmérica.

[11] Odriozola-González P., Planchuelo-Gómez Á., Irurtia M.J., de Luis-García R. (2020). Psychological effects of the COVID-19 outbreak and lockdown among students and workers of a Spanish university. Psychiatry Res..

[12] Gao W., Ping S., Liu X. (2020). Gender differences in depression, anxiety, and stress among college students: A longitudinal study from China. J. Affect. Disord..

[13] Belinchón-deMiguel P., Tornero-Aguilera J.F., Dalamitros A.A., Nikolaidis P.T., Rosemann T., Knechtle B., Clemaleste-Suárez V.J. (2020). Multidisciplinary analysis of differences between finisher and non-finisher ultra-endurance mountain athletes. Front. Physiol..

[14] Sharkey T., Whatnall M.C., Hutchesson M.J., Haslam R.L., Bezzina A., Collins C.E., Ashton L.M. (2020). Effectiveness of gender-targeted versus gender-neutral interventions aimed at improving dietary intake, physical activity and/or overweight/obesity in young adults (aged 17–35 years): A systematic review and meta-analysis. Nutr. J..

[15] Soh N.L., Walter G., Baur L., Collins C. (2009). Nutrition, mood and behaviour: A review. Acta Neuropsychiatr..

[16] Clemente-Suárez V.J., Dalamitros A.A., Beltran-Velasco A.I., Mielgo-Ayuso J., Tornero-Aguilera J.F. (2020). Social and psychophysiological consequences of the COVID-19 pandemic: An extensive literature review. Front. Psychol..

[17] Práxedes A., Moreno A., Sevil J., Del Villar F., García-González L. (2016). Niveles de Actividad Física en Estudiantes Universitarios: Diferencias en Función del Género, la Edad y los Estados de Cambio. Revista Iberoamericana De Psicología Del Ejercicio Y Ei Deporte.

[18] Lores A.P., Murcia J.A.M. (2008). Actitud de los universitarios ante la práctica físico-deportiva: Diferencias por géneros. Rev. Psicol. Deporte.

[19] Rammstedt B., John O.P. (2007). Measuring personality in one minute or less: A 10-item short version of the Big Five Inventory in English and German. J. Res. Personal..

[20] Van Knippenberg F.C.E., Duivenvoorden H.J., Bonke B., Passchier J. (1990). Shortening the state-trait anxiety inventory. J. Clin. Epidemiol..

[21] Bond F.W., Hayes S.C., Baer R.A., Carpenter K.M., Guenole N., Orcutt H.K., Waltz T., Zettle R.D. (2011). Preliminary psychometric properties of the Acceptance and Action Questionnaire–II: A revised measure of psychological inflexibility and experiential avoidance. Behav. Ther..

[22] Russell D.W. (1996). UCLA Loneliness Scale (Version 3): Reliability, validity, and factor structure. J. Personal. Assess..

[23] Zung W.W. (1965). A self-rating depression scale. Arch. Gen. Psychiatry.

[24] Sandín B., Valiente R.M., García-Escalera J., Chorot P. (2020). Impacto psicológico de la pandemia de COVID-19: Efectos negativos y positivos en población española asociados al periodo de confinamiento nacional. Rev. Psicopatología Psicol. Clínica.

[25] Matud M.P. (2004). Gender differences in stress and coping styles. Personal. Individ. Differ..

[26] Pine D.S., Mogg K., Bradley B.P., Montgomery L., Monk C.S., McClure E., Guyer A., Ernst M., Charney D., Kaufman J. (2005). Attention bias to threat in maltreated children: Implications for vulnerability to stress-related psychopathology. Am. J. Psychiatry.

[27] Cabanach R.G., Fariña F., Freire C., González P., del Mar Ferradás M. (2015). Diferencias en el afrontamiento del estrés en estudiantes universitarios hombres y mujeres. Eur. J. Educ. Psychol..

[28] Pacheco N., Durán A., Rey L. (2007). Inteligencia emocional y su relación con los niveles de burnout, engagement y estrés en estudiantes universitarios. Rev. Educ..

[29] Redondo-Flórez L., Tornero-Aguilera J.F., Ramos-Campo D.J., Clemente-Suárez V.J. (2020). Gender Differences in Stress-and Burnout-Related Factors of University Professors. Biomed. Res. Int..

[30] Castañeiras C., Posada M.C. (2006). Estilos de personalidad y su relación con medidas de ansiedad y depresión: Datos normativos para el Inventario MIPS en adultos marplatenses. Rev. Iberoam. Diagnóstico Evaluación-Avaliação Psicológica.

[31] Baker A.H., Wardle J. (2003). Sex differences in fruit and vegetable intake in older adults. Appetite.

[32] Varì R., Scazzocchio B., Del Papa S. (2017). Dietary habits and gender differences. Ital. J. Gend. Specif. Med..

[33] Barzegari A., Ebrahimi M., Azizi M., Ranjbar K. (2011). A study of nutrition knowledge, attitudes and food habits of college students. World Appl. Sci. J..

[34] Yahia N., Wang D., Rapley M., Dey R. (2016). Assessment of weight status, dietary habits and beliefs, physical activity, and nutritional knowledge among university students. Perspect. Public Health.

[35] Moreno C.O.S., Lomelí D.G., Valencia D.G.G. (2020). Actividad física y hábitos alimenticios en universitarios durante la pandemia por COVID-19. Memorias de Extenso.

[36] Tachalov V.V., Orekhova L.Y., Kudryavtseva T.V., Isaeva E.R., Loboda E.S. (2016). Manifestations of personal characteristics in individual oral care. EPMA J..

[37] Alkan A., Cakmak O., Yilmaz S., Cebi T., Gurgan C. (2015). Relationship between psychological factors and oral health status and behaviours. Oral Health Prev. Dent..

[38] Redondo-Flórez L., Fernández-Lucas J., Clemente-Suárez V.J. (2020). Cultural differences in stress-related psychological, nutrition, physical activity and oral health factors of professors. Nutrients.

[39] Von Bothmer M.I., Fridlund B. (2005). Gender differences in health habits and in motivation for a healthy lifestyle among Swedish university students. Nurs. Health Sci..

[40] Clemente F.M., Nikolaidis P.T., Martins F.M.L., Mendes R.S. (2016). Physical activity patterns in university students: Do they follow the public health guidelines?. PLoS ONE.

[41] Bebetsos E., Antoniou P. (2008). University students’ differences on attitudes towards computer use. Comparison with students’ attitudes towards physical activity. Interact. Educ. Multimed..

[42] Valenzuela M.C.S., Gallegos L.I.F., Baca L.R.L., López H.L.M., Rico F.J.F. (2021). Estrés académico en universitarios y la práctica de ejercicio físico-deportivo. Rev. Publicando.

[43] Goenaga A.N.M., Marín A.R. (2020). Factores asociados a los estilos de vida en los estudiantes universitarios. Una aplicación del instrumento fantástico. Rev. Digit. Act. Física Deporte.

